# Psychological Distress, Daytime Sleepiness, Fatigue, and Health-Related Quality of Life in Young Adults: A Cross-Sectional Study with Implications for Rehabilitation

**DOI:** 10.3390/healthcare14213573

**Published:** 2026-10-22

**Authors:** Ahmed A. Torad, Sulaiman Alanazi, Reda Fathi Elbosaty, Noran Abdallah Essa, Omar Hasan-Hafez, András Attila Horváth, Ali Boolani, Harshvardhan Singh, Mona Mohamed Taha, Rana Alawafi, Ahmed M. El Melhat, Mohamed A. Torad, Tamer Mohamed El-Saeed, Mohamed Abdelaziz Emam

**Affiliations:** 1 Department of Physical Therapy and Health Rehabilitation, College of Applied Medical Sciences, Jouf University, Sakaka 72388, Saudi Arabia; sualanazi@ju.edu.sa; 2 Department of Basic Sciences for Physical Therapy, Faculty of Physical Therapy, Kafrelsheikh University, Kafrelsheikh 33511, Egypt; redafathi015@gmail.com (R.F.E.); noraneisa36@gmail.com (N.A.E.); 3 Peconic Bay Medical Center, Riverhead, NY 11901, USA; ohasanhafez@northwell.edu; 4 Neurocognitive Research Centre, Nyírő Gyula National Institute of Psychiatry and Addictology, 1135 Budapest, Hungary; andras.harvath.semmelweis@gmail.com; 5 Department of Anatomy, Histology and Embryology, Semmelweis University, 1085 Budapest, Hungary; 6 Human Performance and Nutrition Research Institute, Oklahoma State University, Stillwater, OK 74078, USA; ali.boolani@okstate.edu (A.B.); singh.harshvardhan@okstate.edu (H.S.); 7 Department of Rehabilitation Sciences, College of Health and Rehabilitation Sciences, Princess Nourah bint Abdulrahman University, P.O. Box 84428, Riyadh 11671, Saudi Arabia; momtaha@pnu.edu.sa (M.M.T.); roalwafi@pnu.edu.sa (R.A.); 8 Department of Physical Therapy for Musculoskeletal Disorders and Its Surgery, Faculty of Physical Therapy, Cairo University, Giza 3750210, Egypt; ahmed.elmelhat@cu.edu.eg; 9 Department of Physical Therapy, Faculty of Health Sciences, Beirut Arab University, Beirut 11072809, Lebanon; 10 Department of Electronics and Communication, Faculty of Engineering, High Technological Institute, 10th of Ramadan City 44629, Egypt; mohamed.torad@hti.edu.eg; 11 Department of Physical Therapy, Faculty of Medical Rehabilitation Sciences, King Abdulaziz University, Jeddah 22254, Saudi Arabia; 12 Department of Physical Therapy for Pediatrics, Faculty of Physical Therapy, Cairo University, Giza 12613, Egypt; 13 János Szentágothai Neurosciences Division, Semmelweis University, 1085 Budapest, Hungary

**Keywords:** fatigue, depression, anxiety, stress, psychological, sleepiness, quality of life

## Abstract

**Highlights:**

**What are the main findings?**

- Psychological distress strongly explained variance in mental and physical fatigue. Depression and anxiety selectively predicted mental and physical health.
- Depression and anxiety selectively predicted mental and physical health.

**What are the implications of the main findings?**

- Rehabilitation screening should evaluate fatigue alongside psychological distress.
- Interventions must distinguish mental from physical fatigue profiles.

**Abstract:**

Background/Objectives: This study examined associations between psychological distress, daytime sleepiness, mental and physical fatigue, and HRQoL in young adults and identified key predictors relevant for rehabilitation-oriented screening and intervention. Methods: In this cross-sectional analytical study, 371 university students (18–25 years) completed the Epworth Sleepiness Scale, Depression Anxiety Stress Scales-21, Fatigue Assessment Scale, and 12-Item Short Form Health Survey. Descriptive statistics, one-way ANOVA, multivariate general linear models, multiple linear regression, and logistic regression were applied to evaluate associations with fatigue and SF-12 physical and mental health scores and categories. Results: Most participants reported mild–moderate distress and fatigue, and about half of the participants had SF-12 scores below the sample median. Severe mental and physical fatigue were associated with substantially higher stress, anxiety, and depression than normal fatigue (all *p* < 0.001). Psychological variables explained 52% of the variance in mental fatigue and 30% in physical fatigue, with lower R^2^ for SF-12 physical (0.17) and mental health (0.29). Depression and stress were the strongest predictors of mental fatigue and low SF-12 mental health scores, whereas anxiety was more closely related to physical fatigue and physical health. Sleepiness showed weak, inconsistent associations after adjustment. Higher distress categories significantly increased the odds of HRQoL below the sample median. Conclusions: Psychological distress, particularly depression and stress, was strongly associated with fatigue and was also associated with poorer health-related quality of life, although the adjusted models indicated a more modest contribution to HRQoL. The findings support the value of considering psychological distress in rehabilitation-oriented screening while recognizing that HRQoL is influenced by multiple factors.

## 1. Introduction

Fatigue, psychological distress, and impaired health-related quality of life (HRQoL) are important concerns among young adults, particularly university students exposed to academic, social, and career-related demands [1,2]. Psychological distress may be accompanied by fatigue and reduced participation in daily activities, making the early identification of these problems relevant to rehabilitation-oriented health assessment [3,4].

Daytime sleepiness may also contribute to fatigue and impaired functioning in young adults. The Epworth Sleepiness Scale (ESS) is widely used to assess subjective daytime sleepiness and has demonstrated good reliability and utility in clinical and non-clinical populations [5,6]. Excessive daytime sleepiness has been associated with poorer cognitive performance, academic functioning, and mental health in young people [7,8]. However, its contribution to fatigue and HRQoL when considered alongside psychological distress remains less clear.

Psychological distress is commonly assessed using the Depression Anxiety Stress Scales-21 (DASS-21), which separately evaluates stress, anxiety, and depression [9,10]. Previous research has shown that higher psychological distress is associated with greater fatigue and poorer HRQoL, with depression frequently showing a strong relationship with these outcomes [4,11,12]. Importantly, fatigue is multidimensional and may include distinct mental and physical components [13,14]. The Fatigue Assessment Scale (FAS) allows these components to be evaluated separately, while the 12-Item Short Form Health Survey (SF-12) provides measures of physical and mental health-related quality of life [15,16].

Although previous studies have examined psychological distress, fatigue, sleep-related problems, and HRQoL, limited evidence has evaluated these domains simultaneously in otherwise healthy young adults. It remains unclear whether psychological distress is more strongly associated with mental and physical fatigue than daytime sleepiness and whether specific psychological domains show different patterns of association with physical and mental HRQoL. Addressing this gap may help identify the most relevant domains for rehabilitation-oriented screening and early support in young adult populations [17,18,19,20].

Existing research in rehabilitation and neurology has shown that combinations of fatigue, depression, and anxiety are associated with quality of life in conditions such as multiple sclerosis, chronic obstructive pulmonary disease, and post-stroke populations [19,20,21]. However, young adults in educational settings often lack access to structured rehabilitation services, even though they may experience substantial psychosocial and fatigue-related burden that could benefit from rehabilitation-oriented interventions, such as activity pacing, exercise programs, and psychological or sleep-focused self-management [4,22]. Clarifying which aspects of psychological distress and sleepiness are the most strongly linked to mental and physical fatigue and to low HRQoL can support efficient screening, triage, and early intervention strategies in these settings.

The current cross-sectional study therefore aims to examine the associations between psychological distress (stress, anxiety, and depression), daytime sleepiness, mental and physical fatigue, and SF-12 physical and mental health in a sample of young adults. Specifically, the objectives are: (1) to determine how stress, anxiety, and depression scores are associated with mental and physical fatigue and whether these associations are stronger for mental than for physical fatigue; (2) to evaluate the associations of stress, anxiety, and depression with SF-12 physical and mental health scores; (3) to examine whether higher categorical levels of psychological distress are associated with increased odds of being classified as having low overall quality of life; and (4) to assess the association between daytime sleepiness and both mental and physical fatigue alongside psychological distress. By addressing these objectives using multivariate general linear and regression models, this study seeks to identify psychological and sleep-related factors associated with fatigue and HRQoL that may inform rehabilitation-oriented screening and guide future research on early supportive strategies in young adult populations.

## 2. Materials and Methods

### 2.1. Study Design and Setting

This cross-sectional analytical study was conducted among university students aged 18–25 years. Participants were recruited from Physical Therapy Faculty, Kafrelsheikh University, Egypt, between June 2024 and June 2026. This study was conducted within the university academic setting, and eligible students at different academic levels were invited to participate. Data were collected during regular academic days using a standardized self-administered questionnaire. After receiving information about this study and providing written informed consent, participants completed the Epworth Sleepiness Scale, Depression Anxiety Stress Scales-21, Fatigue Assessment Scale, and 12-Item Short Form Health Survey. The questionnaires were completed individually in a quiet setting, and participants were allowed sufficient time to complete all measures. Completed questionnaires were checked for completeness before data entry and statistical analysis.

Data were collected in a single assessment session in an educational setting [23], using standardized self-report questionnaires administered in a controlled, quiet environment. The Strengthening the Reporting of Observational Studies in Epidemiology [24] statement guided the planning and reporting of methodological procedures.

### 2.2. Participants

Participants were undergraduate students from the Faculty of Physical Therapy, Kafrelsheikh University, Egypt, aged 18–25 years. Participants were eligible for inclusion if they were between 18 and 25 years of age, could read and understand the study language, and were able to complete the self-administered questionnaires independently. In addition, they were required to report no history of diagnosed psychiatric disorders or chronic medical conditions, such as neurological, cardiovascular, or endocrine diseases, that could substantially influence fatigue or quality of life. Exclusion criteria comprised the current use of psychotropic medication or ongoing treatment for mental health problems, self-reported chronic illness or disability requiring regular medical follow-up, and incomplete questionnaire data on any of the key study variables.

The final sample comprised 371 undergraduate students from the Faculty of Physical Therapy, Kafrelsheikh University, Egypt, aged 18–25 years. The mean age was 19.94 ± 1.02 years. The sample included 273 males (73.6%) and 98 females (26.4%). The mean height, weight, and body mass index (BMI) were 173.99 ± 9.01 cm, 72.65 ± 11.26 kg, and 23.97 ± 3.10 kg/m^2^, respectively. All participants met the predefined eligibility criteria and provided complete data for the study variables.

### 2.3. Recruitment and Sample Size

Participants were recruited through announcements in lectures, posters on campus, and digital invitations distributed via institutional communication channels. Interested students were invited to a designated room where eligibility was checked, and if eligible, written informed consent was obtained before participation. The final sample comprised 371 young adults. There were no incomplete responses as all questions were mandatory for submission. Thus, the conventional minimum recommendations were exceeded (e.g., 10–15 participants per predictor) for stable multivariable regression and logistic regression models in observational rehabilitation research.

### 2.4. Measures

All instruments used are widely applied and display acceptable reliability and validity in diverse populations, including young adults and student samples.

Demographic and anthropometric data were collected.

Participants self-reported age and gender and provided height and weight, which were used to calculate body mass index (BMI; kg/m^2^).

#### 2.4.1. Daytime Sleepiness

Daytime sleepiness was assessed with the Epworth Sleepiness Scale (ESS), an 8-item questionnaire that measures the likelihood of dozing in common situations, scored from 0 to 24, with higher scores indicating greater sleepiness [6,25]. The ESS has demonstrated good internal consistency and test–retest reliability and is widely used in both clinical and non-clinical populations. In this study, ESS scores were analyzed as a continuous variable and categorized as normal (0–10), mild (11–14), moderate (15–18), and severe (>18) for descriptive and logistic regression analyses.

#### 2.4.2. Psychological Distress

Psychological distress was measured using the Depression Anxiety Stress Scales-21 (DASS-21), which consists of three 7-item subscales for depression, anxiety, and stress, rated on a 4-point Likert scale (0–3). Subscale scores were summed and multiplied by two to obtain final scores, which were interpreted using established severity bands (normal, mild, moderate, severe, extremely severe) [10,26]. The DASS-21 has shown good internal consistency, factorial validity, and criterion validity in clinical and community samples, including student and young adult populations [27]. In the present study, depression, anxiety, and stress scores were treated as both continuous predictors and categorical variables in regression analyses.

#### 2.4.3. Fatigue

Fatigue was assessed using the Fatigue Assessment Scale (FAS), a 10-item questionnaire. Items are rated on a 5-point response scale, with higher total scores indicating greater fatigue. The scoring procedure and fatigue severity categories were determined according to the validated version of the instrument used in this study [13,28]. In this study, separate scores for mental fatigue, physical fatigue, and total fatigue were calculated, and fatigue categories (normal, mild, severe) were used to examine group differences in psychological variables.

#### 2.4.4. Health-Related Quality of Life

Health-related quality of life (HRQoL) was measured using the 12-Item Short Form Health Survey (SF-12), which yields two component scores: the Physical Component Summary (PCS; physical health) and Mental Component Summary (MCS; mental health). The SF-12 is a widely used generic HRQoL instrument with well-documented reliability and validity in community and student samples [16,29]. In this study, SF-12 PCS and MCS scores were calculated according to standard scoring procedures, and an overall SF-12 quality-of-life score was also derived. For logistic regression, PCS, MCS, and overall SF-12 scores were dichotomized into “high” and “low” HRQoL based on median splits, which is a common approach in epidemiological rehabilitation research when established cut-offs are not available.

For the logistic regression analyses, SF-12 physical and mental health scores were dichotomized using the sample median as the cut-off to distinguish relatively lower from relatively higher HRQoL within the study sample. These categories represent sample-relative classifications rather than established clinical thresholds. Although dichotomization facilitates binary comparisons, it may reduce information and statistical power compared with analyses using continuous scores, but it was used here to provide clinically interpretable group comparisons and to estimate the likelihood of having relatively poor versus better health-related quality of life.

### 2.5. Procedure

Potential participants received written information about the study objectives and procedures and were given the opportunity to ask questions before providing written informed consent. Data collection took place in small groups in a quiet classroom or laboratory, with a researcher present to provide clarification while ensuring that participants completed the questionnaires independently to minimize interviewer bias. The questionnaire pack included demographic and anthropometric items followed by the ESS, DASS-21, FAS, and SF-12, presented in a standardized order. Completion time was approximately 20–30 min. No incentives were provided, and participants were informed that participation was voluntary and that they could withdraw at any time without consequences.

### 2.6. Ethical Considerations

The study protocol was reviewed and approved by the institutional research ethics committee of the host university (approval number: KFSIRB200-267). All procedures complied with the Declaration of Helsinki and national regulations on research involving human participants. Additional informed consent was obtained from all individual participants for whom identifying information is included in this article. Only aggregated data are reported, and individual participants cannot be identified from the published results.

### 2.7. Statistical Analysis

All analyses were conducted using IBM SPSS Statistics (version 29; IBM Corp., Armonk, NY, USA). All statistical tests were two-tailed, and a *p* value of less than 0.05 was considered statistically significant, in line with common practice in rehabilitation and quality-of-life research [4,14].

Continuous variables, including age, height, weight, body mass index (BMI), Epworth Sleepiness Scale (ESS) scores, Depression Anxiety Stress Scales-21 (DASS-21) subscale scores, Fatigue Assessment Scale (FAS) mental and physical fatigue scores, and Short-Form-12 (SF-12) physical and mental health scores, were summarized using means and standard deviations. Categorical variables such as gender, categories of sleepiness, stress, anxiety, depression, fatigue severity, and health-related quality of life (high vs. low SF-12 physical, mental, and overall quality-of-life categories) were summarized as frequencies and percentages.

To explore differences in psychological and fatigue variables across fatigue severity groups, one-way analyses of variance (ANOVAs) were conducted with fatigue categories (normal, mild, severe) as the independent factor and continuous psychological variables (stress, anxiety, depression, sleepiness) as dependent variables. When overall F-tests were significant, post hoc pairwise comparisons (e.g., Tukey or Bonferroni) were used to identify specific differences between fatigue categories, which is consistent with established approaches in fatigue and quality-of-life studies.

Similarly, ANOVAs were used to compare psychological distress and sleepiness scores across categories of SF-12 physical and mental health (high vs. low health), with follow-up comparisons where appropriate. Effect sizes for group differences were reported using partial eta squared, in line with recommendations for rehabilitation research on fatigue and quality of life.

To address the multivariate nature of the outcomes, general linear models (GLMs) with multiple dependent variables were applied. A multivariate GLM was first estimated with mental fatigue, physical fatigue, SF-12 physical health, and SF-12 mental health as dependent variables and continuous psychological predictors (stress, anxiety, depression, and sleepiness scores) as independent variables. This approach allowed for the simultaneous testing of the impact of psychological distress and sleepiness on multiple correlated outcomes, which is recommended when modeling fatigue and quality of life together [4,19].

First, the overall multivariate effect was evaluated using the multivariate GLM. Significant multivariate effects were followed by univariate tests for each dependent variable, with F-statistics, degrees of freedom, *p* values, and partial eta squared reported. Separate multiple linear regression models were then used to estimate the independent associations of daytime sleepiness, stress, anxiety, and depression with each continuous outcome.

For each dependent variable within the multivariate GLM, F-statistics, corresponding *p* values, partial eta squared (as an effect size index), and observed power were reported. These parameters provided information about the strength and clinical relevance of the associations between psychological factors and each outcome, consistent with multivariate reporting standards in rehabilitation and psychosomatic research.

To quantify the predictive contribution of psychological distress and sleepiness to each continuous outcome, separate multiple linear regression models were constructed. Four models were specified, with mental fatigue, physical fatigue, SF-12 physical health, and SF-12 mental health scores as dependent variables and sleepiness, stress, anxiety, and depression scores as independent variables. For each model, the multiple correlation coefficient (R), coefficient of determination (R^2^), adjusted R^2^, and standard error of the estimate were reported, aligning with analytical practices in rehabilitation and quality-of-life research on fatigue. Multicollinearity among the continuous predictors included in the multiple linear regression models was assessed using tolerance and variance inflation factor (VIF) values. Tolerance values greater than 0.20 and VIF values below 5 were considered indicative of no substantial multicollinearity.

No formal a priori power calculation was performed before recruitment. A sensitivity analysis was therefore conducted based on the final sample size and the four predictors included in the multiple linear regression models: daytime sleepiness, stress, anxiety, and depression. With 371 participants and α = 0.05, this study had 80% power to detect an effect size of approximately f^2^ = 0.038 or larger (R^2^ ≈ 0.037), indicating adequate sensitivity to detect small overall associations.

Unstandardized regression coefficients (B) for each predictor were presented as part of regression equations, enabling the interpretation of how a one-unit increase in each psychological variable was associated with changes in fatigue and quality-of-life scores. This approach mirrors previous work that has used multivariable linear models to identify predictors of fatigue and health-related quality of life in chronic and rehabilitation populations [4,12,21].

To examine the associations between psychological factors and categorical quality-of-life outcomes, binary logistic regression analyses were conducted with low SF-12 physical health and low SF-12 mental health scores as the modeled outcomes. Categorical predictors included the levels of daytime sleepiness, stress, anxiety, and depression, coded using dummy variables, with the severe category specified as the reference category for each predictor. Adjusted odds ratios (ORs) and their 95% confidence intervals (CIs) were calculated for each predictor category relative to its reference category.

Model fit was assessed using −2 log likelihood, Cox–Snell and Nagelkerke pseudo-R^2^ values, the omnibus test, and the Hosmer–Lemeshow goodness-of-fit test. These statistics were used to evaluate overall model performance and goodness of fit.

## 3. Results

The final sample comprised 371 young adults, including 273 males (73.6%) and 98 females (26.4%), with a mean age of 19.94 ± 1.02 years. The mean BMI was 23.97 ± 3.10 kg/m^2^. Most participants reported mild to moderate psychological distress and mild fatigue, while 52.8% were classified as having low overall SF-12 quality of life. Most participants reported mild to moderate psychological distress and mild fatigue, while around half were classified as having low overall quality of life based on SF-12 scores, as shown in Table 1.

Participants with severe fatigue reported markedly higher stress, anxiety, and depression scores than those with normal fatigue, with medium effect sizes, particularly for mental fatigue. Also, young adults classified as having low physical or low mental health scores on the SF-12 had significantly higher distress scores than those with high health scores, whereas differences in sleepiness were small and mostly non-significant, as shown in Table 2.

In the multivariate general linear model, psychological predictors showed a statistically significant overall effect on mental fatigue, with an F value of 5.83, a *p* value less than 0.001, and a partial eta squared of 0.064, indicating a small-to-moderate effect size with excellent observed power (1.00). For physical fatigue, the model was also significant (F = 2.32, *p* = 0.018), with a smaller partial eta squared of 0.026 and good observed power [29], suggesting a modest but reliable multivariate effect on this outcome. In contrast, the multivariate effects on SF-12 physical and mental health scores did not reach statistical significance (physical health: F = 1.95, *p* = 0.102, partial eta squared = 0.022, power = 0.59; mental health: F = 1.62, *p* = 0.170, partial eta squared = 0.019, power = 0.50), indicating weaker and statistically non-significant overall associations with these quality-of-life components at the multivariate level.

For the univariate follow-up tests, the psychological predictors were significantly associated with mental fatigue (F = 5.83, *p* < 0.001, partial η^2^ = 0.064) and physical fatigue (F = 2.32, *p* = 0.018, partial η^2^ = 0.026). The corresponding effects for SF-12 physical health (F = 1.95, *p* = 0.102, partial η^2^ = 0.022) and SF-12 mental health (F = 1.62, *p* = 0.170, partial η^2^ = 0.019) were not statistically significant.

Separate multiple linear regression models were then used to examine the independent associations of daytime sleepiness, stress, anxiety, and depression with each continuous outcome. The models explained 29.6% of the variance in physical fatigue (R^2^ = 0.296; adjusted R^2^ = 0.288), 52.2% in mental fatigue (R^2^ = 0.522; adjusted R^2^ = 0.517), 17.3% in SF-12 physical health (R^2^ = 0.173; adjusted R^2^ = 0.164), and 29.1% in SF-12 mental health (R^2^ = 0.291; adjusted R^2^ = 0.283). Depression and stress showed the strongest associations with mental fatigue and mental health, whereas anxiety showed stronger associations with physical fatigue and physical health. These findings indicate associations rather than causal effects, as shown in Table 3.

Additional regression statistics are presented in Supplementary Tables S1–S3. Multicollinearity diagnostics for the continuous predictors showed tolerance values ranging from 0.316 to 0.859 and VIF values ranging from 1.164 to 3.163, indicating no substantial multicollinearity among daytime sleepiness, stress, anxiety, and depression (Supplementary Table S1). Detailed linear regression estimates, including standardized coefficients, 95% confidence intervals, and squared semi-partial correlations, are presented in Supplementary Table S2. Depression accounted for the largest unique proportion of variance in mental fatigue (sr^2^ = 0.130) and SF-12 mental health (sr^2^ = 0.050), whereas anxiety accounted for the largest unique proportion of variance in physical fatigue (sr^2^ = 0.026) and SF-12 physical health (sr^2^ = 0.033). Adjusted odds ratios and their 95% confidence intervals for low SF-12 physical and mental health scores are presented in Supplementary Table S3. Although both logistic models were statistically significant overall, none of the individual category contrasts were significant in the physical health model. In the mental health model, stress and depression were significant as overall categorical predictors, whereas sleepiness and anxiety were not. The category-specific estimates did not demonstrate a consistent monotonic pattern and should be interpreted cautiously because some categories contained few participants and produced wide confidence intervals.

## 4. Discussion

In this sample of 371 young adults (mean age ≈ 20 years), most participants fell in the mild–moderate range for stress, anxiety, and depression, and the majority reported at least mild fatigue; approximately half were classified as having low overall SF-12 quality of life. These figures indicate a substantial psychosocial and fatigue burden in a non-clinical university population, suggesting that many students may already be functioning close to the threshold where rehabilitation-oriented support (e.g., psychoeducation, activity pacing, basic psychological interventions) is relevant. Elevated distress and fatigue in this age group are biologically and contextually plausible, as the chronic activation of stress systems, irregular sleep, high cognitive load, and reduced physical activity can disrupt neuroendocrine regulation, increase allostatic load, and manifest as persistent tiredness and poorer self-rated health [2,23,30].

Several studies agree that high levels of psychological distress, fatigue, and impaired quality of life are common among university students and emerging adults, with prevalence estimates for anxiety and depressive symptoms often exceeding 25–30% [2,17,31]. Recent work in Saudi and European student samples likewise reports that over half of students experience clinically relevant fatigue and that distress and academic load are key correlates, consistent with the current findings [4,32]. Some studies during the COVID-19 period reported even higher distress and fatigue levels than observed here, particularly when online learning disruptions and financial stressors are prominent, suggesting that contextual events may further amplify the burden in specific cohorts [33,34]. Conversely, a minority of investigations have found more favorable mental health profiles in students with strong social support, adequate sleep, and lower academic demands, indicating that the burden seen in this study is not inevitable and may be modifiable through targeted environmental and behavioral interventions [7,18].

Participants with severe physical and especially severe mental fatigue had markedly higher stress, anxiety, and depression scores than those with normal fatigue [35], with medium effect sizes and highly significant differences across categories. Mean distress scores increased stepwise from normal through mild to severe fatigue, and post hoc tests showed that the largest differences were between the normal and severe fatigue categories, particularly for stress and depression. This gradient indicates a graded association between higher psychological distress and greater perceived fatigue, consistent with neurobiological models linking affective dysregulation, attentional bias, and central fatigue [13,36].

Numerous studies support a positive association between higher psychological distress and higher fatigue levels among university students and in rehabilitation populations [37]. For example, a large student survey during COVID-19 found that higher psychological distress was strongly correlated with perceived fatigue and reduced learning capacity [34], while research in multiple sclerosis and stroke has repeatedly demonstrated that depression and anxiety are robust correlates of fatigue, independent of physical disability [4,11,12]. Recent work among undergraduates similarly reports that higher perceived stress and irregular sleep are associated with more frequent and severe fatigue complaints, echoing the pattern observed in the present study [32]. However, some longitudinal analyses in clinical cohorts have suggested that the directionality may be more complex, with fatigue sometimes mediating the effect of depression on quality of life rather than simply being its consequence [38]. These findings do not contradict the current cross-sectional gradients but highlight that in rehabilitation contexts, fatigue and psychological distress may be related over time, although the direction and temporal sequence of these associations cannot be determined from the present cross-sectional data.

Our findings should also be considered in the context of research conducted specifically among university students and healthy young adults. Studies in these populations have reported meaningful associations between psychological distress, sleep-related problems, fatigue, and health-related quality of life, supporting the relevance of these factors during early adulthood [39]. Evidence from university and young adult populations may provide a more appropriate comparison for the present findings than studies conducted primarily in patients with chronic diseases or rehabilitation populations. Accordingly, the interpretation of the present results emphasizes evidence from student and young adult populations where available, while the findings from clinical populations are considered complementary rather than directly comparable evidence.

In the multivariate models, psychological predictors accounted for a substantial proportion of the variance in mental fatigue (R^2^ ≈ 0.52) and a moderate proportion of the variance in physical fatigue (R^2^ ≈ 0.30), whereas their explanatory contribution to SF-12 physical and mental health was more modest (R^2^ ≈ 0.17 and 0.29, respectively; adjusted R^2^ ≈ 0.16 and 0.28). These findings suggest that psychological distress was more consistently associated with fatigue than with health-related quality of life. Although depression, stress, and anxiety showed associations with SF-12 outcomes, the adjusted models provide a more cautious interpretation because the predictors were considered simultaneously and their independent contributions were less pronounced than the overall bivariate and unadjusted associations might suggest. In particular, the relatively modest, accounted-for variance for physical health indicates that factors beyond psychological distress are likely to contribute substantially to perceived physical quality of life in this young adult sample. Similarly, although the mental health model showed a larger proportion of variance, the results should not be interpreted as demonstrating that psychological distress is independently associated with HRQoL. Rather, the findings indicate that psychological distress is one of several factors associated with perceived health status. The cross-sectional design also prevents conclusions regarding temporal or causal relationships between psychological distress and quality of life.

These findings are broadly consistent with rehabilitation and psychosomatic research reporting associations between depression, stress, fatigue, and mental health-related quality of life (HRQoL), while anxiety has also been associated with physical symptoms and functioning. However, these patterns vary across populations and study designs, and the present cross-sectional findings do not establish causal pathways or confirm that any psychological factor is an independent determinant of fatigue or HRQoL. Longitudinal studies in multiple sclerosis and other chronic conditions indicate that depression affects HRQoL largely through fatigue, particularly mental fatigue [35,36]. Similarly, studies among university students have identified stress and depression as the main predictors of poorer mental HRQoL, whereas anxiety is closely associated with somatic complaints and reduced physical activity [17,18]. However, some studies have reported anxiety as the strongest predictor of both mental and physical HRQoL, particularly among medical students and populations experiencing high academic stress, highlighting the impact of contextual factors such as academic field, gender, and coping strategies [32,37].

Participants whose SF-12 physical or mental health scores fell below the sample median had significantly higher stress, anxiety, and depression scores than those whose scores were at or above the median, whereas ESS sleepiness differed little between groups. These categories reflect relative differences within the study sample rather than established clinical classifications. The adjusted logistic regression analyses showed that associations between psychological distress and HRQoL below the sample median were not consistent across all categories. Although some individual category comparisons were statistically significant, others were not, indicating that the observed associations depended on the specific predictor category and outcome. These findings should be interpreted cautiously, given the cross-sectional design and the categorization of continuous psychological measures.

Several studies in both students and clinical populations corroborate the strong link between psychological distress and HRQoL below the sample median, often showing that depression and stress account for more variance in physical and mental health than sleep parameters alone [2,12,21]. Path-analytical work in neurological rehabilitation has even suggested that the effect of depression on HRQoL may be largely mediated by fatigue, reinforcing the central role of emotional factors in perceived health [38,40]. On the other hand, some investigations in medical and nursing students have reported stronger and more direct associations between ESS-measured sleepiness and quality of life than observed here, sometimes with a clear dose–response pattern, indicating that in cohorts with more pronounced sleep deprivation, sleepiness may play a larger role [41,42]. The comparatively modest contribution of sleepiness in the present study may therefore reflect relatively moderate ESS scores and the absence of severe sleep deprivation, suggesting that psychological distress and fatigue may warrant particular attention during rehabilitation-oriented assessment, while sleep-related behaviors should also be considered. However, these findings should not be interpreted as establishing a hierarchy of treatment priorities or demonstrating that interventions targeting psychological distress will improve fatigue or HRQoL.

Although the observed associations between psychological distress, daytime sleepiness, fatigue, and health-related quality of life are clinically relevant, several potentially important factors were not assessed in the present study. In particular, sleep quality, physical activity, substance use, academic workload, and social support may influence psychological distress and quality-of-life outcomes and may therefore have contributed to the observed associations. For example, poor sleep quality and insufficient physical activity may be associated with greater fatigue, daytime sleepiness, psychological distress, and poorer health-related quality of life. Similarly, academic demands and limited social support may affect psychological well-being and perceived quality of life among university students, while substance use may represent an additional behavioral factor associated with psychological distress and sleep-related outcomes. Because these variables were not measured or included in the analyses, their potential confounding effects could not be evaluated. Therefore, the observed associations should be interpreted cautiously and cannot be assumed to represent independent relationships after accounting for these unmeasured factors. Future studies should incorporate these variables into the study design and multivariable analyses to provide a more comprehensive assessment of the factors associated with psychological distress, fatigue, daytime sleepiness, and health-related quality of life.

This study has several methodological limitations. First, its cross-sectional design precludes causal inference and does not establish the direction of the observed associations among psychological distress, daytime sleepiness, fatigue, and health-related quality of life (HRQoL). Second, participants were recruited from a single university, and the sample was predominantly male (73.6%). These characteristics may limit the generalizability of the findings to other universities, non-student populations, and populations with a more balanced sex distribution. Third, all study variables were assessed using self-report questionnaires, without an objective verification of sleep, physical activity, or health status. Consequently, the findings may be affected by recall bias, social desirability bias, and common-method variance. Fourth, several potentially relevant confounding factors, including physical activity, diet, substance use, sleep quality, academic workload, and social support, were not assessed or included in the analyses. These factors may have influenced the observed associations. Finally, administering the questionnaires in a standardized order may have introduced order effects. These limitations should be considered when interpreting the results, and the findings should be generalized cautiously beyond populations comparable to the study sample. Future studies involving more diverse populations, objective measurements, and longitudinal designs may help clarify the observed associations and improve the generalizability of the findings.

Additionally, dichotomizing SF-12 scores using a median split may be associated with some loss of information and reduced statistical power compared with modeling the scores as continuous outcomes. Therefore, the logistic regression findings should be interpreted as associations with relatively lower versus higher health-related quality of life within this sample rather than as effects based on established clinical thresholds.

Implications for rehabilitation practice. The present findings may have several practical implications for rehabilitation-oriented assessment and early intervention in young adults. Because psychological variables explained a substantial proportion of the variance in mental fatigue and a moderate proportion of the variance in physical fatigue, the rehabilitation assessment of young adults presenting with persistent fatigue may benefit from considering psychological distress alongside fatigue severity rather than assessing fatigue in isolation. A brief multidimensional screening of stress, anxiety, depression, and mental and physical fatigue may help identify different symptom profiles and guide appropriate referral and management. For example, individuals presenting predominantly with mental fatigue and elevated depressive or stress symptoms may benefit from psychoeducation, psychological support or referral, and strategies addressing cognitive and emotional load, whereas those with greater physical fatigue may require an assessment of activity tolerance, participation, physical activity patterns, and the potential need for activity pacing or a structured physical activity program. Sleep-related behaviors and daytime sleepiness may also be considered complementary components of assessment, although the relatively weaker contribution of sleepiness in the adjusted models suggests that it should not necessarily be the primary rehabilitation target in this population. Such multidimensional assessment may help characterize symptom profiles and identify individuals who may benefit from further assessment, appropriate referral, or supportive care. Sleep-related behaviors and daytime sleepiness may also be considered as complementary assessment domains. Nevertheless, these implications should be interpreted as potential applications of the observed associations, not as evidence of causal mechanisms, independent determinants, or established treatment effectiveness. Because this study was cross-sectional and did not evaluate any rehabilitation intervention, longitudinal studies and controlled intervention trials are needed to determine whether targeting psychological distress improves fatigue or HRQoL.

## 5. Conclusions

In conclusion, psychological distress, particularly depression and stress, was strongly associated with fatigue and was also associated with poorer health-related quality of life, although adjusted associations varied across categories. These findings support considering psychological distress in rehabilitation-oriented screening while recognizing that HRQoL is multifactorial. Given the cross-sectional design, causal relationships cannot be inferred. Future longitudinal and multicenter studies using objective measures of sleep and physical activity are needed to confirm these associations and improve generalizability.

## Figures and Tables

**Table 1:** Demographic characteristics, daytime sleepiness, psychological distress, fatigue, and health-related quality of life of participants (*n* = 371).

Variable	Category	Mean ± SD
Age ^a^ (years)		19.94 ± 1.02
Gender	Male	273 (73.6%)
	Female	98 (26.4%)
Height (cm)		173.99 ± 9.01
Weight (kg)		72.65 ± 11.26
BMI ^b^ (kg/m^2^)		23.97 ± 3.10
Epworth Sleepiness Scale score		8.19 ± 4.19
Sleepiness category (ESS) ^c^	Normal	261 (70.4%)
	Mild	91 (24.5%)
	Moderate	15 (4.0%)
	Severe	4 (1.1%)
Stress score (DASS-21) ^d^		17.93 ± 9.23
Stress category (DASS-21)	Normal	84 (22.6%)
	Mild	126 (34.0%)
	Moderate	103 (27.8%)
	Severe	40 (10.8%)
	Extremely severe	18 (4.9%)
Anxiety score (DASS-21)		13.86 ± 9.05
Anxiety category (DASS-21)	Normal	91 (24.5%)
	Mild	31 (8.4%)
	Moderate	107 (28.8%)
	Severe	47 (12.7%)
	Extremely severe	95 (25.6%)
Depression score (DASS-21)		15.42 ± 9.13
Depression category (DASS-21)	Normal	91 (24.5%)
	Mild	72 (19.4%)
	Moderate	114 (30.7%)
	Severe	47 (12.7%)
	Extremely severe	47 (12.7%)
Mental fatigue score (FAS) ^e^		13.02 ± 4.73
Mental fatigue category (FAS)	Normal	130 (35.0%)
	Mild	176 (47.4%)
	Severe	65 (17.5%)
Physical fatigue score (FAS)		13.75 ± 3.33
Physical fatigue category (FAS)	Normal	62 (16.7%)
	Mild	261 (70.4%)
	Severe	48 (12.9%)
Total fatigue score (FAS)		26.77 ± 7.00
Overall fatigue category	Normal	82 (22.1%)
	Mild	237 (63.9%)
	Severe	52 (14.0%)
SF-12 ^f^ physical health score		53.54 ± 19.87
SF-12 physical health category	High	220 (59.3%)
	Low	151 (40.7%)
SF-12 mental health score		47.50 ± 18.04
SF-12 mental health category	High	158 (42.6%)
	Low	213 (57.4%)
SF-12 overall quality-of-life score		50.52 ± 16.28
SF-12 overall quality-of-life category	High	175 (47.2%)
	Low	196 (52.8%)

^a^ = chronological age of participants in years; ^b^ = body mass index; ^c^ = Epworth Sleepiness Scale; ^d^ = Depression Anxiety Stress Scales-21; ^e^ = Fatigue Assessment Scale; ^f^ = 12-Item Short Form Health Survey.

**Table 2:** Comparisons of daytime sleepiness and psychological distress scores across fatigue severity and health-related quality-of-life categories.

Variable	Category	Sleepiness	Stress	Anxiety	Depression
Physical Fatigue ^a^	Normal	7.21 ± 3.65	13.13 ± 7.75	8.35 ± 6.58	10.32 ± 7.28
	Mild	8.21 ± 4.41	17.23 ± 8.18	13.3 ± 7.81	14.65 ± 8.13
	Severe	9.38 ± 3.31	27.96 ± 9.28	24.04 ± 10.16	26.17 ± 8.11
MD (Normal–Mild)		1.001	4.097	4.940	4.329
*p* value		0.236	<0.001	<0.001	<0.001
MD (Normal–Severe)		−2.165	−14.829	−15.687	−15.844
*p* value		0.016	<0.001	<0.001	<0.001
MD (Mild–Severe)		−1.164	−10.732	−10.747	−11.515
*p* value		0.197	<0.001	<0.001	<0.001
Mental Fatigue ^b^	Normal	6.5 ± 4.25	12.17 ± 7.82	9.37 ± 6.81	9.15 ± 6.58
	Mild	8.89 ± 3.95	19 ± 7.35	14.48 ± 7.95	16.09 ± 7.17
	Severe	9.71 ± 3.63	26.55 ± 8.66	21.17 ± 10.55	26.12 ± 7.52
MD (Normal–Mild)		2.707	6.509	7.335	8.095
*p* value		0.003	<0.001	<0.001	<0.001
MD (Normal–Severe)		−3.538 *	−10.204	−7.913	−14.165
*p* value		0.002	<0.001	<0.001	<0.001
MD (Mild–Severe)		−0.831	−3.695	−0.578	−6.07
*p* value		1.000	0.127	1.000	0.001
SF−12 ^e^ Physical Health ^c^	High	7.76 ± 4.06	15.85 ± 8.34	11.52 ± 7.72	13.27 ± 8.26
	Low	8.82 ± 4.32	20.95 ± 9.64	17.27 ± 9.76	18.54 ± 9.46
Mean Difference		0.266	3.234	2.893	1.196
ANOVA ^f^ F		0.125	5.693	4.344	0.933
*p* value		0.724	0.018	0.038	0.335
SF-12 Mental Health ^d^	High	7.19 ± 4.2	13.51 ± 7.6	10.65 ± 7.51	10.8 ± 7.14
	Low	8.94 ± 4.04	21.21 ± 8.97	16.24 ± 9.37	18.85 ± 8.94
Mean Difference		−0.543	−4.389	−2.812	−5.909
ANOVA F		0.493	9.880	3.869	21.497
*p* value		0.483	0.002	0.050	<0.001

^a^ Physical fatigue categories were based on the Fatigue Assessment Scale (FAS). ^b^ Mental fatigue categories were based on the Fatigue Assessment Scale (FAS). ^c^ Physical health categories were derived from the SF-12 Physical Component Summary score. ^d^ Mental health categories were derived from the SF-12 Mental Component Summary score. ^e^ = 12-Item Short Form Health Survey; ^f^ = analysis of variance; SF-12; * = Statistically Significant.

**Table 3:** Multiple linear and logistic regression analyses with fatigue and SF-12 health outcomes in young adults.

Outcome	R/−2LL ^a^	R^2^/Cox–Snell R^2 b^	Adj. R^2^/Nagelkerke R^2 c^	Model Equation
Physical fatigue	0.544	0.296	0.288	10.631 − 0.043·Sleepiness + 0.047·Stress + 0.093·Anxiety + 0.088·Depression
Mental fatigue	0.723	0.522	0.517	6.277 + 0.094·Sleepiness + 0.092·Stress − 0.012·Anxiety + 0.291·Depression
SF-12 ^d^ physical health	0.416	0.173	0.164	SF-12 physical health = 69.033 − 0.268(Sleepiness) − 0.016(Stress) − 0.632(Anxiety) − 0.314(Depression)
SF-12 mental health	0.539	0.291	0.283	SF-12 mental health = 67.714 − 0.382(Sleepiness) − 0.363(Stress) + 0.003(Anxiety) − 0.689(Depression)
Binary logistic regression				
Low SF-12 physical health scores (cat) ^f^	446.365	0.138	0.186	logit(*p*) ^e^ = −0.406 + 0.350·Sleepiness_mild + 0.504·Sleepiness_moderate − 0.027·Sleepiness_severe + 0.991·Stress_mild − 0.350·Stress_moderate + 0.225·Stress_severe + 0.192·Stress_extreme + 0.375·Anxiety_mild + 0.037·Anxiety_moderate − 0.263·Anxiety_severe − 0.631·Anxiety_extreme + 0.797·Depression_mild + 0.274·Depression_moderate − 0.081·Depression_severe − 0.488·Depression_extreme
Low SF-12 mental health scores (cat)	405.878	0.237	0.318	logit(*p*) = 3.379 − 0.354·Sleepiness_mild + 0.063·Sleepiness_moderate − 0.421·Sleepiness_severe − 1.571·Stress_mild − 1.988·Stress_moderate − 1.493·Stress_severe − 2.523·Stress_extreme − 0.761·Anxiety_mild − 0.454·Anxiety_moderate − 0.332·Anxiety_severe − 0.292·Anxiety_extreme + 1.988·Depression_mild − 0.865·Depression_moderate − 0.332·Depression_severe − 1.318·Depression_extreme

^a^ = the multiple correlation coefficient for linear regression models, whereas −2LL (−2 log likelihood) is reported for binary logistic regression models; ^b^ = the coefficient of determination for multiple linear regression models, whereas Cox–Snell R^2^ is reported for binary logistic regression models; ^c^ Adjusted R^2^ = multiple linear regression models, whereas Nagelkerke R^2^ is reported for binary logistic regression models; ^d^=SF-12 = 12-Item Short Form Health Survey; ^e^ = the natural logarithm of the odds, which estimate the probability of belonging to the group with low health-related quality of life using the logit transformation; ^f^ = categorical outcome (low vs. high SF-12 health scores).

## Data Availability

The datasets used and/or analyzed during the current study are available from the corresponding authors on reasonable request due to ethical restrictions.

## Supplementary Materials

The following supporting information can be downloaded at https://www.mdpi.com/article/10.3390/healthcare14213573/s1, Table S1: Multicollinearity diagnostics for predictors included in the multiple linear regression models. Table S2: Detailed multiple linear regression results for fatigue and SF-12 health outcomes. Table S3: Adjusted odds ratios for low SF-12 physical and mental health scores. Table S4: A STROBE-based reporting checklist for a cross-sectional study.

## Abbreviations

The following abbreviations are used in this manuscript:

ANOVA	Analysis of Variance
BMI	Body Mass Index
DASS-21	Depression Anxiety Stress Scales-21
ESS	Epworth Sleepiness Scale
FAS	Fatigue Assessment Scale
GLM	General Linear Model
HRQoL	Health-Related Quality of Life
MCS	Mental Component Summary
PCS	Physical Component Summary
SD	Standard Deviation
SF-12	12-Item Short Form Health Survey
SPSS	Statistical Package for the Social Sciences
STROBE	Strengthening the Reporting of Observational Studies in Epidemiology
