# Supplementary material for: Psychological Distress, Daytime Sleepiness, Fatigue, and Health-Related Quality of Life in Young Adults: A Cross-Sectional Study with Implications for Rehabilitation
Source: Healthcare (Basel). 2026 Oct 22;14(21):3573. doi: 10.3390/healthcare14213573 (PMC13762800; doi:10.3390/healthcare14213573)
Supplement: Supplementary file 1 [file healthcare-14-03573-s001.zip › healthcare-4626559-supplementary.pdf]

Supplementary Table S1

Table S1. Multicollinearity diagnostics for predictors included in the multiple linear regression models

**Predictor**	**Tolerance**	**VIF**
Daytime sleepiness	0.859	1.164
Stress	0.316	3.163
Anxiety	0.406	2.465
Depression	0.411	2.434

Note: VIF, variance inflation factor. The four multiple linear regression models included daytime sleepiness, stress, anxiety, and depression as continuous predictors. The same tolerance and VIF values were obtained for the physical fatigue, mental fatigue, SF-12 physical health, and SF-12 mental health models because the same predictors and analytical sample were used. Tolerance values ranged from 0.316 to 0.859, and VIF values ranged from 1.164 to 3.163, providing no indication of substantial multicollinearity based on the prespecified criteria of tolerance greater than 0.20 and VIF less than 5. Values are based on the current SPSS output, which included 371 participants.

Supplementary Table S2

Table S2. Detailed multiple linear regression results for fatigue and SF-12 health outcomes

Physical fatigue

Predictor	B	SE	Standardized β	95% CI for B	t	p	Unique variance, sr^2
Constant	10.631	0.379	—	9.885 to 11.377	28.014	<0.001	—
Daytime sleepiness	−0.043	0.038	−0.055	−0.117 to 0.030	−1.158	0.248	0.003
Stress	0.047	0.028	0.129	−0.009 to 0.102	1.657	0.098	0.005
Anxiety	0.093	0.025	0.254	0.043 to 0.143	3.684	<0.001	0.026
Depression	0.088	0.025	0.241	0.039 to 0.137	3.519	<0.001	0.024

Model statistics: R^2=0.296; adjusted R^2=0.288; F(4,366)=38.408, p<0.001.

Mental fatigue

Predictor	B	SE	Standardized β	95% CI for B	t	p	Unique variance, sr^2
Constant	6.277	0.445	—	5.402 to 7.152	14.114	<0.001	—
Daytime sleepiness	0.094	0.044	0.084	0.008 to 0.181	2.144	0.033	0.006
Stress	0.092	0.033	0.179	0.027 to 0.157	2.785	0.006	0.010
Anxiety	−0.012	0.030	−0.022	−0.070 to 0.047	−0.394	0.694	<0.001
Depression	0.291	0.029	0.561	0.233 to 0.348	9.953	<0.001	0.130

Model statistics: R^2=0.522; adjusted R^2=0.517; F(4,366)=99.948, p<0.001.

SF-12 physical health

Predictor	B	SE	Standardized β	95% CI for B	t	p	Unique variance, sr^2
Constant	69.033	2.458	—	64.200 to 73.867	28.085	<0.001	—
Daytime sleepiness	−0.268	0.243	−0.057	−0.746 to 0.210	−1.102	0.271	0.003

Predictor	B	SE	Standardized β	95% CI for B	t	p	Unique variance, sr^2
Stress	0.016	0.182	0.008	−0.342 to 0.374	0.090	0.929	<0.001
Anxiety	−0.632	0.164	−0.288	−0.954 to −0.310	−3.856	<0.001	0.033
Depression	−0.314	0.161	−0.144	−0.631 to 0.004	−1.942	0.053	0.008

Model statistics: R^2=0.173; adjusted R^2=0.164; F(4,366)=19.126, p<0.001.

SF-12 mental health

Predictor	B	SE	Standardized β	95% CI for B	t	p	Unique variance, sr^2
Constant	67.714	2.066	—	63.651 to 71.778	32.771	<0.001	—
Daytime sleepiness	−0.382	0.204	−0.089	−0.784 to 0.020	−1.869	0.062	0.007
Stress	−0.363	0.153	−0.185	−0.664 to −0.062	−2.369	0.018	0.011
Anxiety	0.003	0.138	0.001	−0.268 to 0.274	0.022	0.983	<0.001

Predictor	B	SE	Standardized β	95% CI for B	t	p	Unique variance, sr^2
Depression	−0.689	0.136	−0.349	−0.956 to −0.422	−5.075	<0.001	0.050

Model statistics: R^2=0.291; adjusted R^2=0.283; $F(4,366)$=37.518, $p<0.001$.

Note: B, unstandardized regression coefficient; SE, standard error; β, standardized regression coefficient; CI, confidence interval; sr^2, squared semi-partial correlation. The sr^2 statistic represents the proportion of outcome variance uniquely associated with a predictor after accounting for the other predictors in the model. It was calculated by squaring the part correlation reported by SPSS. All predictors were entered simultaneously. Values are based on the current SPSS output involving 371 participants.

Supplementary Table S3

Table S3. Adjusted odds ratios for low SF-12 physical and mental health

Low SF-12 physical health

Predictor	Category	B	n	Adjusted OR	95% CI	p
Daytime sleepiness	Mild	0.350	91	1.420	0.116 to 17.415	0.784
	Moderate	0.504	15	1.655	0.109 to 25.049	0.716
	Normal	-0.027	261	0.973	0.081 to 11.641	0.983
	Severe		4	1.000	Reference	—
Stress	Extremely severe	0.991	18	2.695	0.613 to 11.845	0.189
	Mild	-0.350	126	0.705	0.287 to 1.730	0.445
	Moderate	0.225	103	1.252	0.557 to 2.817	0.586
	Normal	0.192	84	1.212	0.398 to 3.691	0.736
	Severe		40	1.000	Reference	—
Anxiety	Extremely severe	0.375	95	1.455	0.661 to 3.201	0.351
	Mild	0.037	31	1.037	0.390 to 2.761	0.941
	Moderate	-0.263	107	0.769	0.367 to 1.613	0.487
	Normal	-0.631	91	0.532	0.224 to 1.262	0.152
	Severe		47	1.000	Reference	—

Predictor	Category	B	n	Adjusted OR	95% CI	p
Depression	Extremely severe	0.797	47	2.220	0.857 to 5.747	0.100
	Mild	0.274	72	1.316	0.562 to 3.078	0.527
	Moderate	-0.081	114	0.922	0.439 to 1.936	0.830
	Normal	-0.488	91	0.614	0.241 to 1.565	0.307
	Severe		47	1.000	Reference	—

Model statistics: -2LL=446.365; Cox–Snell R^2=0.138; Nagelkerke R^2=0.186; omnibus \chi^2(15)=55.043, p<0.001; Hosmer–Lemeshow p=0.789; correct classification, 67.9%.

Low SF-12 mental health

Predictor	Category	B	n	Adjusted OR	95% CI	p
Daytime sleepiness	Mild	-0.354	91	0.702	0.044 to 11.127	0.802
	Moderate	0.063	15	1.065	0.053 to 21.580	0.967
	Normal	-0.421	261	0.656	0.043 to 9.949	0.762
	Severe		4	1.000	Reference	—
Stress	Extremely severe	-1.571	18	0.208	0.024 to 1.797	0.153

Predictor	Category	B	n	Adjusted OR	95% CI	p
	Mild	-1.988	126	0.137	0.035 to 0.532	0.004
	Moderate	-1.493	103	0.225	0.060 to 0.841	0.027
	Normal	-2.523	84	0.080	0.018 to 0.358	0.001
	Severe		40	1.000	Reference	—
Anxiety	Extremely severe	-0.761	95	0.467	0.186 to 1.173	0.105
	Mild	-0.454	31	0.635	0.219 to 1.838	0.402
	Moderate	-0.332	107	0.717	0.316 to 1.627	0.427
	Normal	-0.292	91	0.747	0.303 to 1.841	0.526
	Severe		47	1.000	Reference	—
Depression	Extremely severe	1.988	47	7.304	1.246 to 42.835	0.028

Predictor	Category	B	n	Adjusted OR	95% CI	p
	Mild	-0.865	72	0.421	0.168 to 1.052	0.064
	Moderate	-0.332	114	0.718	0.311 to 1.656	0.437
	Normal	-1.318	91	0.268	0.101 to 0.707	0.008
	Severe		47	1.000	Reference	—

Model statistics: -2LL=405.878; Cox–Snell R^2=0.237; Nagelkerke R^2=0.318; omnibus \chi^2(15)=100.253, p<0.001; Hosmer–Lemeshow p=0.342; correct classification, 71.7%.

Note: OR, odds ratio; CI, confidence interval; SF-12, 12-Item Short Form Health Survey. The modeled event was low SF-12 physical or mental health; high health was coded as the reference outcome. Odds ratios were adjusted simultaneously for sleepiness, stress, anxiety, and depression categories. Severe status was used as the reference category for each predictor in the current SPSS output. The overall categorical effects were p=0.499, p=0.186, p=0.185, and p=0.085 for sleepiness, stress, anxiety, and depression, respectively, in the physical-health model; the corresponding values in the mental-health model were p=0.895, p=0.016, p=0.595, and p=0.002. Wide confidence intervals, particularly for sleepiness, reflect sparse observations in the severe-sleepiness reference category. Values are based on 371 participants.

Supplementary Table S4

STROBE-Based Reporting Checklist for a Cross-Sectional Study

Section/topic	Item	Paraphrased reporting requirement	Status	Where reported in the manuscript
Title and abstract	1a	Identify the study design using a standard term in the title or abstract.	Reported	Title ("A Cross-Sectional Study…") and Abstract, Methods sentence ("In this cross-sectional analytical study…").
Title and abstract	1b	Provide a balanced, informative abstract describing what was done and found.	Reported	Structured Abstract: Background/Objectives, Methods, Results, and Conclusions.
Introduction: background	2	Explain the scientific background and rationale for the investigation.	Reported	Section 1, paragraphs describing fatigue, psychological distress, daytime sleepiness, HRQoL, and their relevance to young adults and rehabilitation.
Introduction: objectives	3	State the specific objectives and any prespecified hypotheses.	Reported	Last paragraph of Section 1, where the study aims/objectives are stated; objectives are also summarized in the Abstract.
Methods: study design	4	Present the key elements of the study design early in the manuscript.	Reported	Section 2.1, first paragraph beginning "This cross-sectional analytical study…".
Methods: setting	5	Describe the setting, location, and relevant recruitment and data-collection dates.	Reported	Section 2.1, first paragraph: Physical Therapy Faculty, Kafr Elsheikh University; university setting; recruitment/data collection from June 2024 to June 2026.
Methods: participants	6a	State eligibility criteria and explain the source and method of participant selection.	Reported	Sections 2.2 (eligibility criteria) and 2.3 (recruitment and sample size); Section 2.1 notes eligible students from different academic levels were invited.
Methods: variables	7	Define outcomes, exposures/predictors, possible confounders, and effect modifiers; provide diagnostic criteria where relevant.	Reported	Section 2.4 and Subsections 2.4.1–2.4.4 define daytime sleepiness, psychological distress, fatigue, and HRQoL; Section 2.7 identifies model predictors and outcomes.
Methods: measurement	8*	For each variable, report its data source and assessment method; describe comparability across groups if relevant.	Reported	Section 2.4 and Subsections 2.4.1–2.4.4: Epworth Sleepiness Scale, DASS-21, Fatigue Assessment Scale, and SF-12; all participants completed the same measures under the same conditions.
Methods: bias	9	Describe measures taken to address potential sources of bias.	Reported	Section 2.1, paragraph beginning "Data were collected in a single assessment session…" and Section 2.5: standardized self-administration, quiet/controlled setting, individual completion, sufficient time, and completeness checks. Residual recall, social-desirability, order, and common-method biases are acknowledged in Section 4.
Methods: study size	10	Explain how the study size was determined.	Reported	Section 2.3, "Recruitment and sample size."
Methods: quantitative variables	11	Explain how quantitative variables were analyzed and, when grouped, justify or define the groupings.	Reported	Subsections 2.4.1–2.4.4 report instrument scoring/categories; Section 2.7 explains use of continuous scores and categories, including the SF-12 median split and statistical analyses.
Methods: statistical methods	12a	Describe statistical methods, including methods used to account for confounding.	Reported	Section 2.7: descriptive statistics, one-way ANOVA, multivariate GLM, multiple linear regression, and logistic regression. Supplementary Tables S1–S3 provide diagnostics and adjusted model estimates.
Methods: statistical methods	12b	Describe methods used for subgroup, category, or interaction analyses.	Reported	Section 2.7: comparisons across fatigue/distress categories and multivariate analyses.
Methods: statistical methods	12c	Explain how missing data were handled.	Reported	Section 2.1 states that completed questionnaires were checked for completeness before data entry. The number with missing responses was reported in 2.3
Methods: statistical methods	12d	If applicable, describe analyses that account for the sampling strategy.	Not applicable	No complex survey, clustered, stratified, or weighted sampling analysis is described; participants were recruited from one university.

Section/topic	Item	Paraphrased reporting requirement	Status	Where reported in the manuscript
Methods: statistical methods	12e	Describe any sensitivity analyses.	Not applicable	No formal sensitivity analysis is reported. Continuous and categorical models are presented as complementary analyses rather than sensitivity analyses.
Results: participants	13a*	Report participant numbers at each relevant study stage and the number analyzed.	Reported	Opening participant-description paragraph in Section 3 and the participant-characteristics table report 371 analyzed participants.
Results: participants	13b*	Give reasons for nonparticipation or exclusion at each relevant stage.	Not applicable / optional	No detailed reasons for refusal/nonparticipation were located in the submitted text.
Results: participants	13c*	Consider presenting participant flow in a diagram.	Not applicable / optional	A flow diagram is optional. The single-session cross-sectional design had no follow-up; add a simple recruitment flow diagram only if screening/exclusion counts are available.
Results: descriptive data	14a*	Report participant characteristics and relevant exposure/predictor and confounder information.	Reported	Section 3, opening descriptive-results paragraphs and the participant/descriptive characteristics table.
Results: descriptive data	14b*	Report the number of participants with missing data for each variable.	Reported	The analyses and Supplementary Tables S1–S3 use 371 participants, The number with missing responses was reported in 2.3.
Results: outcome data	15*	Report outcome-event numbers or appropriate summary measures.	Reported	Section 3 descriptive tables and results for fatigue, psychological distress, daytime sleepiness, and SF-12 physical and mental health outcomes.
Results: main results	16a	Report unadjusted and adjusted estimates when relevant, with precision; identify adjustment variables and rationale.	Reported	Section 3 multivariate/regression results and Supplementary Tables S2–S3. Table S2 gives coefficients and 95% CIs; Table S3 gives adjusted odds ratios and 95% CIs, adjusted simultaneously for sleepiness, stress, anxiety, and depression.
Results: main results	16b	Report cut points when continuous variables are categorized.	Reported	Subsections 2.4.1–2.4.4 and Section 2.7 describe scoring/categories; Supplementary Table S3 reports the predictor categories and reference categories. Section 4 discusses the SF-12 median split.
Results: main results	16c	When relevant, translate relative estimates into absolute risk over a meaningful period.	Not applicable	The study is cross-sectional and does not estimate incidence or risk over time.
Results: other analyses	17	Report other analyses, such as subgroup/category, interaction, or sensitivity analyses.	Reported	Section 3 reports category comparisons and additional multivariate/regression analyses; Supplementary Tables S1–S3 report multicollinearity diagnostics, detailed linear regressions, and adjusted logistic regressions. No interaction or formal sensitivity analysis was conducted.
Discussion: key results	18	Summarize the main findings in relation to the study objectives.	Reported	Opening paragraphs of Section 4 and Section 5.
Discussion: limitations	19	Discuss limitations, including possible sources, direction, and magnitude of bias or imprecision.	Reported	Section 4 paragraph beginning "This study has four key methodological limitations…" and the following paragraph on SF-12 dichotomization.
Discussion: interpretation	20	Interpret findings cautiously in light of objectives, limitations, multiple analyses, and relevant evidence.	Reported	Section 4 throughout, particularly the paragraph beginning "In the multivariate models…" and the limitations paragraphs; causal claims are explicitly avoided.
Discussion: generalisability	21	Discuss the external validity/generalizability of the findings.	Reported	Section 4 limitations paragraph: single university, gender imbalance, student sample, and restriction of generalization to comparable young-adult university populations.
Other information: funding	22	Report the funding source and the funder's role in the study.	Reported	Funding statement identifies Princess Nourah bint Abdulrahman University Researchers Supporting Project (PNURSP2026R209).
